# Fulfilment of patients’ mandatory expectations are crucial for satisfaction: a study amongst 352 patients after total knee arthroplasty (TKA)

**DOI:** 10.1007/s00167-022-07301-y

**Published:** 2023-02-06

**Authors:** Cornelia Lützner, Franziska Beyer, Ludwig David, Jörg Lützner

**Affiliations:** Department for Orthopaedic, Trauma and Plastic Surgery, University Hospital Carl Gustav Carus, TU Dresden, Fetscherstr. 74, 01307 Dresden, Germany

**Keywords:** Total knee arthroplasty, TKA, Expectations, Fulfilment, Satisfaction, Patient-reported outcome measure, PROMs

## Abstract

**Purpose:**

Patient satisfaction with the results of their total knee arthroplasty (TKA) is one of the primary goals of this elective procedure. Furthermore, the association between the fulfilment of patients’ expectations and their satisfaction is well known. The aim of this study was to identify the key expectations of patients awaiting a TKA, evaluate their fulfilment, and compare the outcomes between very and not fully satisfied patients.

**Methods:**

A prospective cohort study of patients with knee OA scheduled for primary TKA was performed. Pre- and one-year postoperatively patient-reported outcome measures (PROMs) were assessed. Expectations and their fulfilment were evaluated via a questionnaire encompassing 31 expectations. Preoperatively, expectations were indicated as mandatory, desirable and not important. Postoperatively, fulfilment was rated as exceeded, fulfilled, partially or not fulfilled, and not applicable. Satisfaction with the results of TKA was measured with a numeric rating scale (NRS) of 0–10. Discrimination between not fully satisfied and very satisfied patients was set at ≥ 8, as has been proposed recently. To identify independent predictors of this discrimination, a multivariate logistic regression analysis was performed.

**Results:**

Complete data sets of 352 patients were analysed. A set of 17 key expectations was identified. Relief of knee pain was fulfilled the most, and improvement of physical function was fulfilled the least. When asked about overall fulfilled expectations, 40% of patients rated them as exceeded, 34% as fulfilled and 26% as less fulfilled than expected. Not fully satisfied patients showed significantly lower PROMs pre- and postoperatively and less fulfilled key expectations. Higher numbers of exceeded and fulfilled mandatory expectations, higher overall fulfilment and better range of motion (ROM) were significant predictors for satisfaction ≥ 8.

**Conclusion:**

Patients’ expectations of TKA outcomes were high with equal emphasis on knee-related and general health-related aspects. Their fulfilment was positively associated with satisfaction. Surgeons should ask patients about mandatory expectations for successful TKA and counsel them about the likelihood of their fulfilment to avoid unrealistic expectations.

**Level of evidence:**

II.

**Supplementary Information:**

The online version contains supplementary material available at 10.1007/s00167-022-07301-y.

## Introduction

Total knee arthroplasty (TKA) is usually the last treatment option in patients with end-stage osteoarthritis of the knee (knee OA) after a long history of different therapies. The decision for this surgery is associated with a number of individual outcome expectations [8, 24, 28, 34, 41]. Regarding relief of pain and functional recovery, TKA is one of the most effective treatments for knee OA [26]. However, studies have shown that patients´ expectations are numerous, not limited to pain and function, and vary depending on patient characteristics, such as gender, age or BMI [8, 18, 21, 24, 28, 41]. Patients’ expectations have been reported as a major factor in the decision-making process in TKA [3]. Consequently, their fulfilment influences postoperative outcome assessment [38]. In particular, growing evidence exists for a strong association between fulfilled expectations and satisfaction with TKA results [15, 18, 28, 33, 39, 40]. In a large cohort study (*n* = 1703), Bourne et al. identified unfulfilled expectations as the strongest contributing variable to patient dissatisfaction after TKA [5].

As largely acknowledged, a considerable number of patients remain not fully satisfied after TKA [10]. Proportions of dissatisfied patients vary greatly, and high numbers of up to 30% have been reported [5–7, 14, 30]. The common understanding is that approximately one in five TKA patients expresses some dissatisfaction after TKA [5]. Therefore, questioning patients about their satisfaction with the results of TKA is an important part of outcome assessment, but there is no gold standard for measuring it [19]. Most commonly, a single question about overall satisfaction with response format either on an ordinal scale or a numeric rating scale (NRS), and respective visual analogue scale (VAS) was applied in TKA studies [19]. To date, there exists no validated cut-off point for discrimination between satisfied and dissatisfied patients of the latter mentioned NRS/VAS 0–10 scale. Most recently, Tolk et al. [36] proposed a NRS satisfaction score of  ≥ 8 (maximum 10) as a discrimination value between very satisfied and not fully satisfied. By applying this cut-off in an explorative investigation amongst a large TKA cohort, the presented study aimed to assess patients’ expectations before TKA and to identify key expectations as well as evaluate fulfilment of these key expectations one year after TKA. Furthermore, differences in PROMs and satisfaction with the results of TKA as well as fulfilment of expectations between very satisfied and not fully satisfied patients were investigated. Finally, the association of fulfilled expectations and postoperative outcomes on discrimination into very satisfied or not fully satisfied was evaluated.

It was hypothesised that patients present high expectations before surgery, but not all would be fulfilled postoperatively. Furthermore, it was hypothesised that greater fulfilment of expectations and better outcomes leads to very satisfied patients.

## Materials and methods

This prospective cohort study has been performed in compliance with the Helsinki Declaration and has been approved by the ethics committee of the TU Dresden (EK 423112014).

Between 09/2017 and 11/2019, all patients with knee OA scheduled for primary TKA surgery in a university hospital were informed about this study and asked to participate. Inclusion criteria were patients with advanced knee OA (grade 3 and 4 Kellgren and Lawrence), primary TKA (no partial arthroplasty or revision surgery), ability to understand German language, and signed informed consent. Patients were handed a set of PROMs including Oxford Knee Score [25], EuroQoL-5D-3L [11], UCLA activity scale [1], and a questionnaire regarding outcome expectations of patients before undergoing TKA surgery [41]. The items of this questionnaire were developed via a 3-stage Delphi study amongst patients with knee OA considering a TKA [21]. The expectation questionnaire consisted of 31 items reflecting symptoms, physical function, physical activity, quality of life, coping strategies, activities of daily life, and various issues, i.e. longevity of implant [41]. Patients were asked for their personal importance of the items in terms of a successful TKA. Possible answers were: mandatory (main goal – needs to be fulfilled to judge the TKA as successful), desirable (secondary goal – fulfilment is not necessary) and not important (not a goal). A study nurse was available to assist in case of problems with completion. Baseline data (age, gender, Body Mass Index (BMI), ASA score, range of motion (ROM)), as well as treatment data (diagnosis, grade of OA, type of implant, X-ray, adverse events, and any re-operations and revision surgeries within 1 year postoperatively) were collected. One year after surgery, patients were invited for clinical examination and filling in of the PROMs. Fulfilment of the same 31 expectations was assessed with the possible answers: exceeded, fulfilled, partially fulfilled, not fulfilled, and not applicable (Supplement 1). Further, a global rating scale was included in which patients indicated their overall fulfilment of expectations on a NRS, with 0 not fulfilled at all, 10 fulfilled exactly as expected, and the range between 10 and 20 fulfilled better than expected. Overall satisfaction with the results of the TKA was evaluated via a NRS (0 very dissatisfied to 10 very satisfied) [7] and patients were asked if they would undergo this surgery again if it was required on the other knee joint. Possible responses were: definitely yes, possibly yes, not sure, probably not, or certainly not [15].

All surgeries were performed by three different surgeons using a medial parapatellar approach without a tourniquet. All implants were cemented and no patellar resurfacing was performed. Full weight-bearing was allowed immediately and patients completed a standardised rehabilitation protocol.

**Fig. 1:**
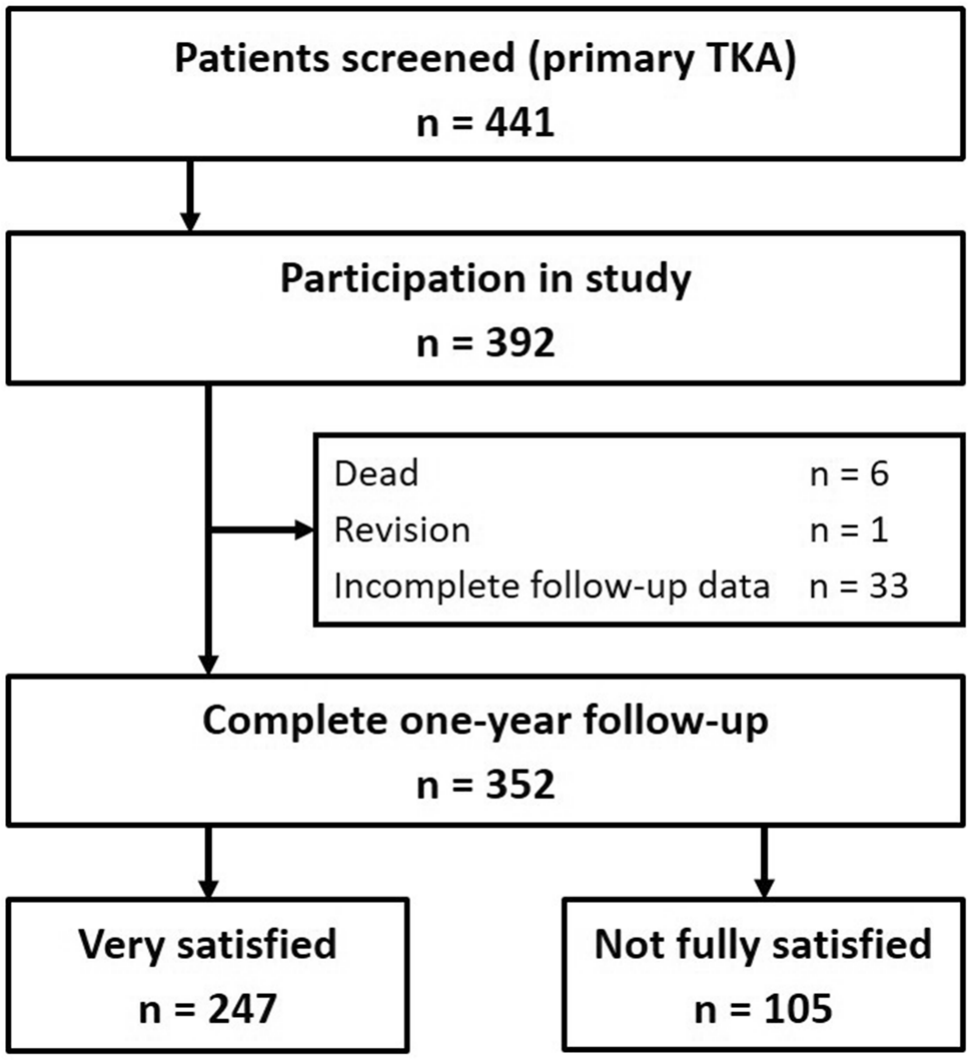
Flow Chart. *TKA* total knee arthroplasty

Between 09/2017 and 11/2019, altogether 441 patients received a primary TKA and 392 participated in this study. Until the one-year follow-up, six patients had died and one revision had occurred due to peri-prosthetic infection whilst undergoing oncological chemotherapy two months after surgery; 33 patients did not complete the follow-up, resulting in 352 complete data sets for analysis (Fig. 1). The mean age of the analysed cohort was 68.8 years (SD 10.0), mean BMI 31.0 kg/m^2^ (SD 5.9), 54.5% were female, and 53.4% had serious comorbidities (ASA score 3 or 4). The majority of 328 patients (93.1%) received a bicondylar TKA and 24 patients (6.9%) needed a rotating-hinge prosthesis in severe valgus deformity.

### Statistical analysis

Sample size calculation was not conducted due to the exploratory design of this prospective study. All statistical analyses were performed using SPSS^®^ software release 27 for Windows (SPSS Inc, Chicago, IL, USA). Data are reported as mean and standard deviation (SD) for continuous values and absolute and relative frequencies for categorical values, respectively. Comparison between time points was performed by paired *t* test for continuous and by McNemar’s respective Wilcoxon signed-rank test for categorical data. Based on the proposed cut-off by Tolk et al. [36], the overall satisfaction scale was used to discriminate between not fully satisfied patients for NRS scores < 8 and very satisfied patients for NRS scores ≥ 8. Group comparison was performed by unpaired *t* test for continuous and by chi-squared respective Mann–Whitney *U* test for categorical data. The significance level was set at *p* < 0.05.

To determine key expectations, a threshold of 75% of patients indicating them as mandatory (main goal) was defined. Most important key expectations were identified by a threshold of 90%. This approach was recently introduced by a study investigating the expectations of patients before undergoing total hip arthroplasty (THA) [22]. Expectations regarding implant longevity were not included in the analysis of fulfilment as it refers to long-time follow-up. Multivariate logistic regression analysis was performed to identify independent predictors (fulfilment of expectations and postoperative outcomes) of discrimination between not fully satisfied and very satisfied patients in a stepwise regression model. As indices for the predictive capacity of the logistic regression model, CoxSnell and Nagelkerke pseudo-*R*^2^ were calculated [4]. CoxSnell *R*^2^ has an upper bound of less than 1.0, whilst Nagelkerke *R*^2^ is an adjusted version of CoxSnell *R*^2^ and ranges between 0 and 1. The higher the *R*^2^ value, the better the fit between the model and the data.

## Results

Within one year after surgery, 12 re-operations were performed (due to one acute peri-prosthetic infection treated with a DAIR procedure, four superficial wound infections, three traumatic capsule ruptures, two hemato-seromas, one patella fracture, and one rupture of the quadriceps tendon). In addition, 11 patients required manipulations under anaesthesia.

**Fig. 2:**
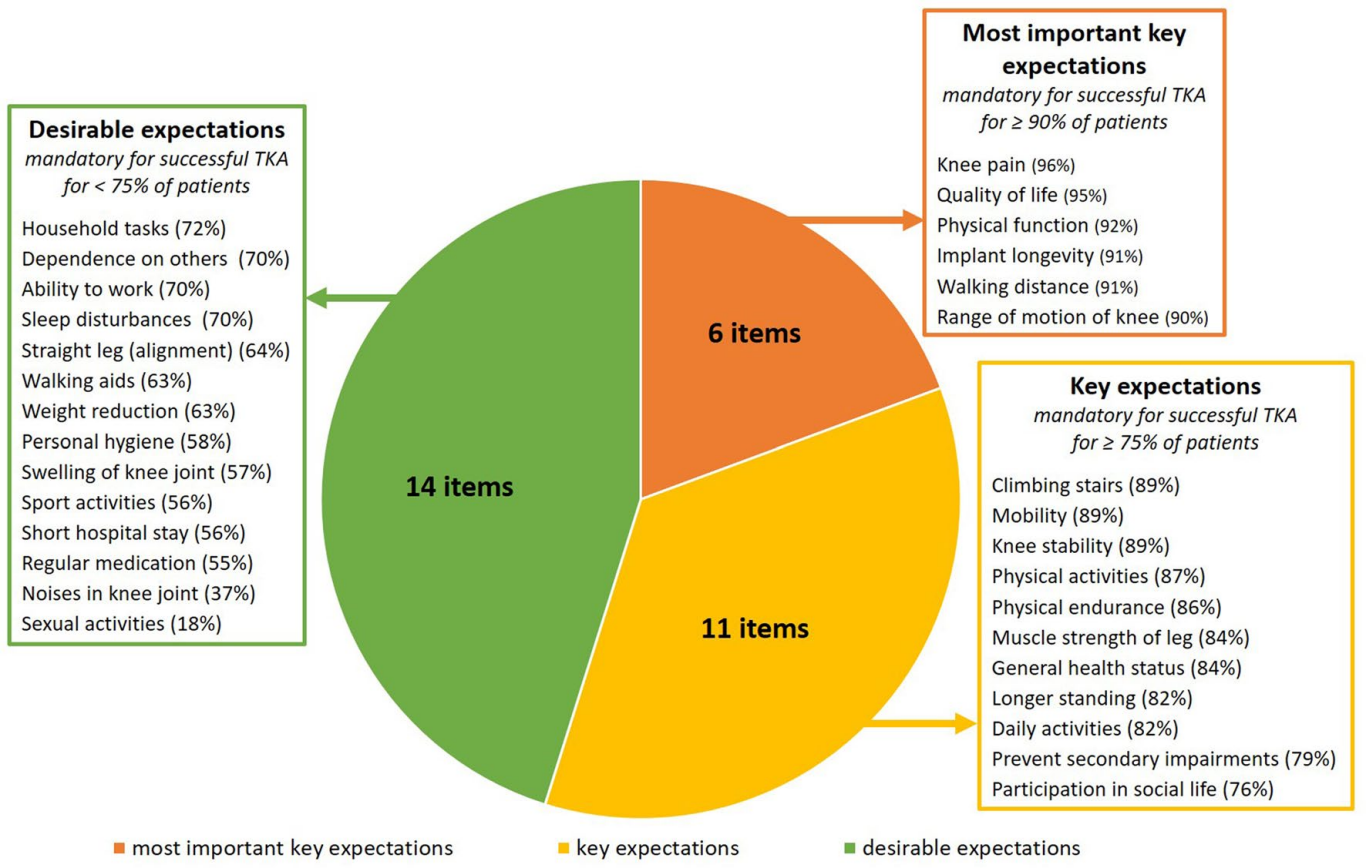
Illustration of mandatory expectations of patients preoperatively. *TKA* total knee arthroplasty

Out of 31 expectations, patients indicated a mean of 23 (SD 5.9) as mandatory (main goal) for a successful TKA and 5 (SD 4.7) as desirable (secondary goal). Six expectations were rated as mandatory by at least 90% of patients (most important key expectations) and another 11 by at least 75% of patients (key expectations) (Fig. 2). The least important expectation was an improvement in sexual activities, which was indicated by 53.4% as no goal.

**Fig. 3:**
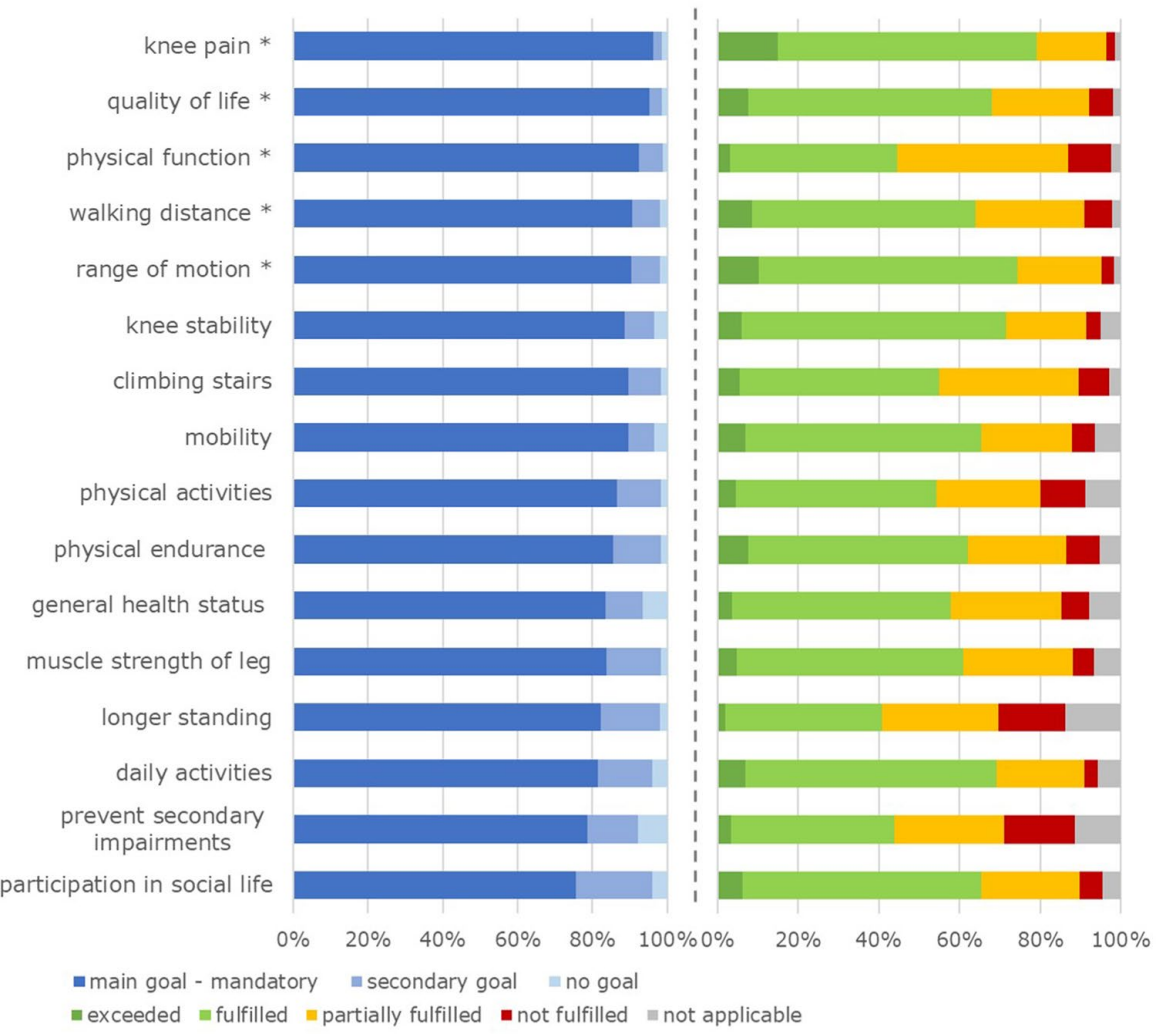
Distribution of preoperative main, secondary, and no goals and fulfilment of 16 key expectations in per cent (without implant longevity), *indicating most important key expectations

Fulfilment of 16 key expectations (without implant longevity) is presented in Fig. 3. Relief of knee pain was fulfilled or exceeded in the largest proportion of patients (64% fulfilled and 15% exceeded). Improvement of physical function was least fulfilled (11% not and 42% only partially fulfilled). Overall fulfilment of expectations on the global rating scale was rated in mean 11.0 (SD 3.7). In 39.9% of patients, fulfilment was better than expected (> 10.0), in 34.4%, it was exactly as expected (= 10.0), and in 25.7%, it was lower than expected (< 10.0).

**Fig. 4:**
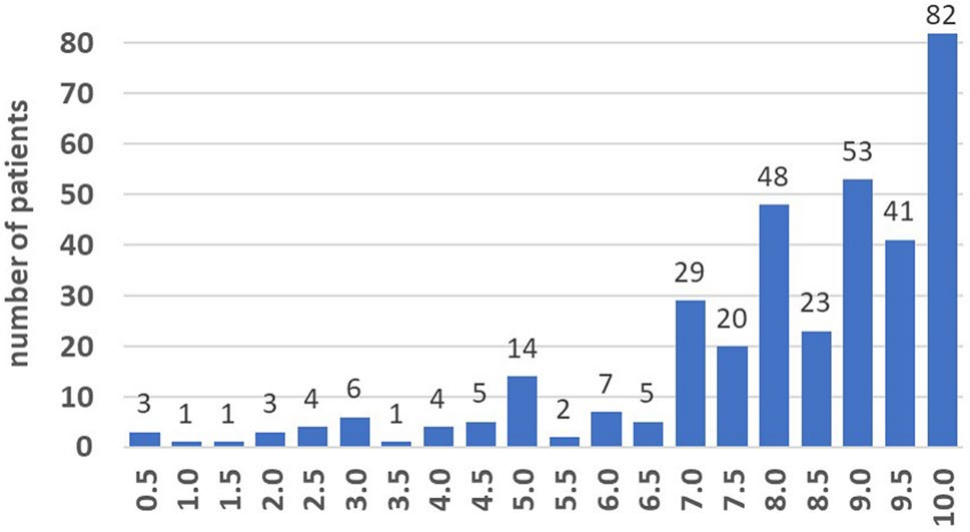
Distribution of answers on the satisfaction NRS 0–10

Satisfaction with the results of surgery was rated a mean of 8.1 (SD 2.1), the distribution of answers on the satisfaction NRS 0–10 is shown in Fig. 4.

247 patients indicated a satisfaction score of  ≥ 8 (70.2%), and 105 patients had a satisfaction score of < 8 (29.8%) on the NRS. Not fully satisfied patients were significantly older, had more comorbidities, had worse ROM postoperatively, and showed significantly lower PROMs (Table 1). The majority of very satisfied patients would undergo TKA surgery again.

**Table 1:** Sociodemographic data and PROMs of not fully satisfied vs. very satisfied patients

Variables (mean, SD)	Not fully satisfied *N *= 105	Very satisfied *N* = 247	*p* value
Age	70.8 (SD 9.7)	67.9 (SD 10.0)	**0.013**
BMI	31.1 (SD 5.7)	30.9 (SD 5.9)	0.782
Female gender	61 (58.1%)	131 (53.0%)	
Male gender	44 (41.9%)	116 (47.0%)	0.383
ASA group 1/2	40 (38.1%)	124 (50.2%)	
ASA group 3/4	65 (61.9%)	123 (49.8%)	**0.037**
OKS (0–48)			
Preoperative	17.7 (SD 6.4)	20.8 (SD 7.3)	** < 0.001**
1-year follow-up	29.6 (SD 7.9)	38.9 (SD 6.7)	** < 0.001**
EuroQol Index (0–1)			
Preoperative	0.54 (SD 0.28)	0.55 (SD 0.28)	0.792
1-year follow-up	0.75 (SD 0.19)	0.87 (SD 0.17)	** < 0.001**
EuroQol VAS (0–100)			
Preoperative	47.7 (SD 17.1)	53.9 (SD 18.8)	**0.003**
1-year follow-up	58.5 (SD 17.9)	73.5 (SD 18.4)	** < 0.001**
UCLA activity scale (0–10) (median, Q1, Q3)			
Preoperative	3.0 (3.0, 4.0)	4.0 (3.0, 6.0)	**0.011**
1-year follow-up	4.0 (3.0, 5.0)	5.0 (4.0, 7.0)	** < 0.001**
Range of motion			
Preoperative	106.2 (SD 17.1)	104.8 (SD 17.3)	0.483
1-year follow-up	108.8 (SD 16.4)	115.2 (SD 11.6)	**0.020**
Leg axis			
Preoperative	− 3.8 (SD 9.6)	− 4.5 (SD 9.0)	0.528
1-year follow-up	− 0.5 (SD 3.3)	− 0.6 (SD 2.7)	0.823
Satisfaction NRS (0–10)	5.6 (SD 1.9)	9.2 (SD 0.7)	** < 0.001**
Surgery again			
Yes	61 (58.1%)	231 (93.5%)	
Uncertain	34 (32.4%)	12 (4.9%)	
No	10 (9.5%)	4 (1.6%)	** < 0.001**

Significant values are marked in bold (*p* > 0.05)

*ASA* American society of anesthesiologists, BMI body-mass-index, *NRS* numeric rating scale, *OKS* oxford knee score, *Q* quartile, *SD* standard deviation, *UCLA* University of California, Los Angeles, *VAS* visual analogue scale

**Fig. 5:**
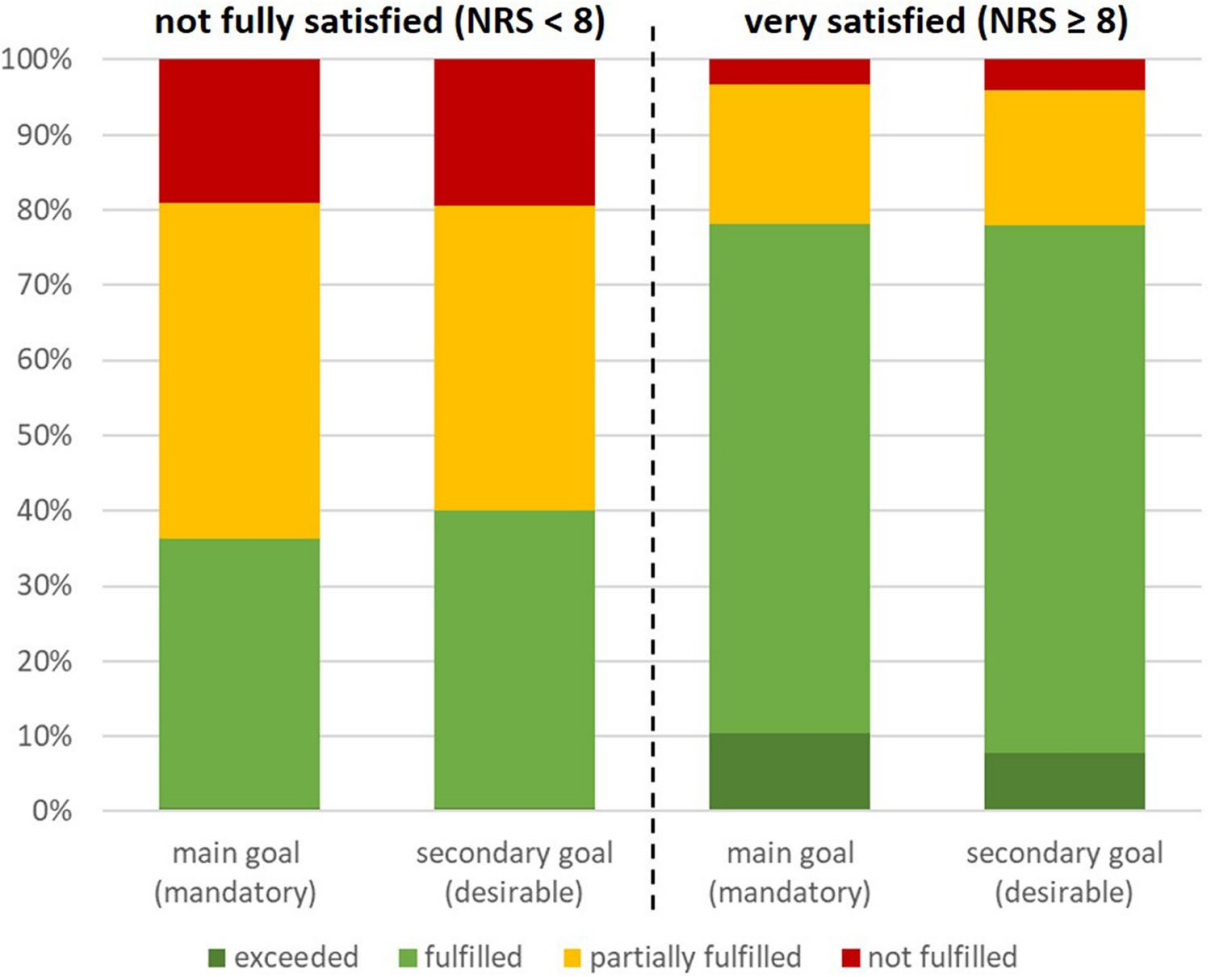
Proportions of exceeded, fulfilled, partially or not fulfilled main and secondary goals of not fully satisfied vs. very satisfied patients. *NRS* numeric rating scale

Preoperative expectations in terms of the number of items indicated as main, secondary, or no goals did not differ between the two groups. Overall fulfilment of expectations was a mean of 12.3 (SD 3.2) in very satisfied and a mean of 8.0 (SD 3.2) in not fully satisfied patients (*p* < 0.001). Proportions of exceeded, fulfilled, partially or not fulfilled main and secondary goals were significantly different (Fig. 5).

**Fig. 6:**
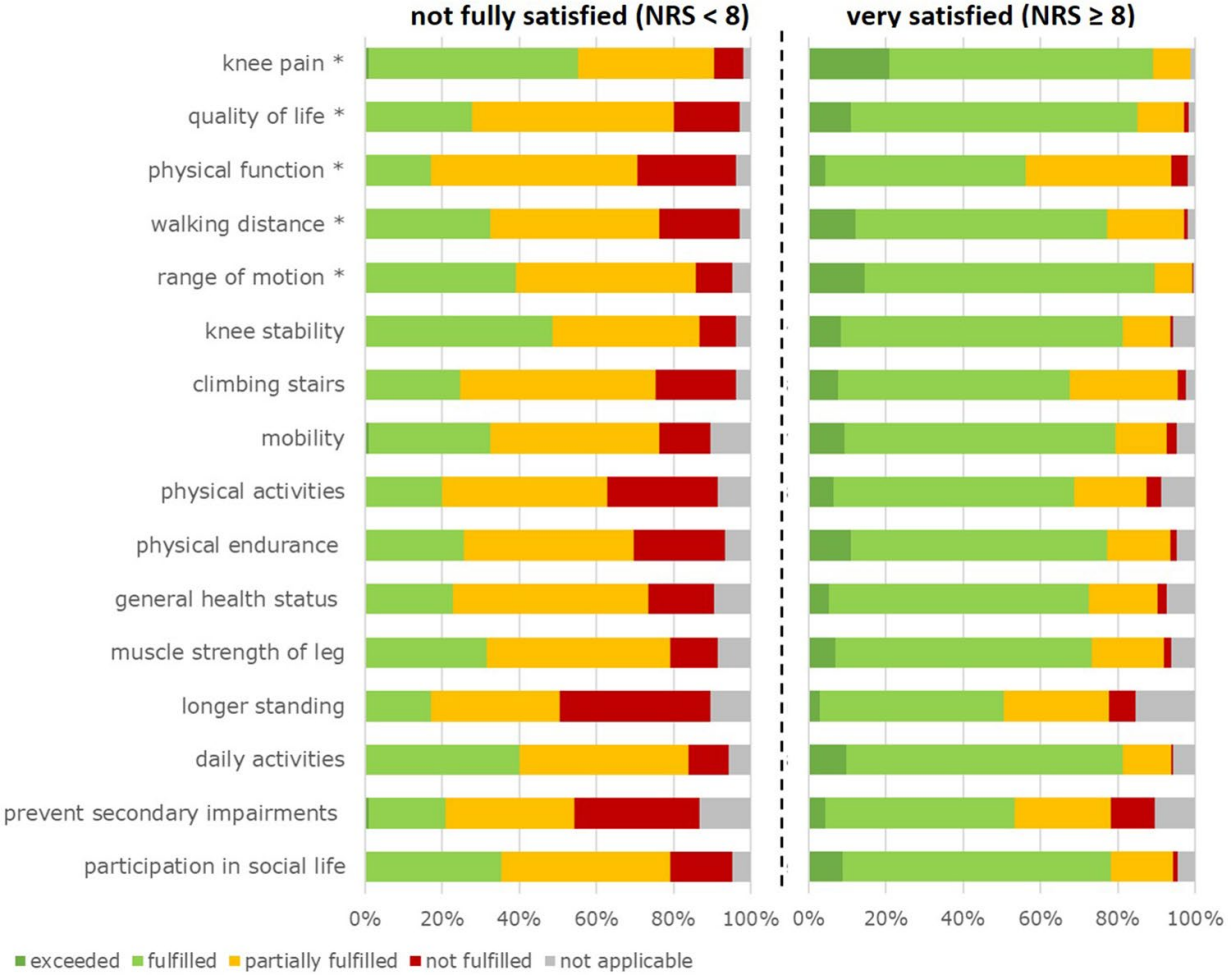
Distribution of fulfilment of 16 key expectations in per cent (without implant longevity) of not fully vs. very satisfied patients *indicating most important key expectations. *NRS* numeric rating scale

Comparison of fulfilled key expectations showed significant differences with a considerably higher proportion of not or partially fulfilled expectations amongst not fully satisfied patients (Fig. 6). In both groups, improvement of physical function was least fulfilled, whereas relief of knee pain was best fulfilled in the not fully satisfied patients and ROM in the very satisfied patients.

In multivariate logistic regression analysis, significant predictors for allocation into the group of very satisfied patients (NRS ≥ 8) were a higher number of exceeded main goals (*p* = 0.031), a higher overall fulfilment of expectations on the global rating scale (*p* = 0.038), a higher number of fulfilled main goals (*p* = 0.002), and better ROM postoperatively (*p* = 0.010). Table 2 shows the final model, which includes the significant predictors only. Interpretation of the odds ratios (Exp(B)) in this model implies that one more exceeded main goal increases the probability of being very satisfied by 180%, an increase by one point of overall fulfilment on the global rating scale by 30%, one more fulfilled main goal by 12%, and an increase by one degree ROM increases the probability by 5%.

**Table 2:** Logistic regression of discrimination between not fully and very satisfied patients

Discrimination between not fully satisfied and very satisfied patients			
Predictors in the model (CoxSnell *R*^2^ = 0.359, Nagelkerke *R*^2^ = 0.528)	B	Exp(B) (95% CI)	*p* value
Numbers of exceeded main goals	1.027	2.791 (1.098; 7.097)	0.031
Overall fulfilment of expectations	0.262	1.300 (1.014; 1.666)	0.038
Numbers of fulfilled main goals	0.115	1.122 (1.043; 1.206)	0.002
ROM postoperatively	0.047	1.048 (1.011; 1.086)	0.010

*ROM* range of motion

## Discussion

The main findings of this study were high outcome expectations with equal importance of knee-related and general health-related aspects, lower fulfilment of knee-related activities and PROMs in not fully satisfied patients, and the positive association of exceeded and fulfilled mandatory expectations on satisfaction.

Patients confirmed high expectations by indicating a mean of 23 out of 31 as mandatory for a successful TKA. Expectations of patients undergoing TKA are generally high [9, 17, 18, 23, 42], but their impact on satisfaction after TKA remains controversial. Many or overly optimistic expectations may contribute to a lower fulfilment rate and result in dissatisfaction. On the other hand, positive health and illness coping behaviour resulting in higher satisfaction may be enhanced [14, 20]. In this study, high expectations were found, but these were not different between not fully satisfied and very satisfied patients.

TKA is highly effective in terms of pain relief and functional recovery [26] and more than 90% of patients indicated these goals as mandatory for a successful TKA. This is consistent with previous studies based on the Hospital for Special Surgery Knee Replacement Expectation Survey (HHS-KRES) [24], where pain relief, walking ability and walking stairs were consistently rated amongst the most important expectations [8, 12, 18, 29, 32]. A recently published study applying the same questionnaire as this study confirmed that reduced knee pain, improved ROM, walking distance, stair walking and overall physical function are mandatory for the majority of patients [41]. In the presented study, quality of life, general health status, participation in social life, prevention of secondary impairments and longevity of the prosthesis were of similar importance to knee pain and function. This is consistent with the published study of Conner-Spady et al., who identified 24 expectations themes in TKA and THA patients, amongst them quality of life, well-being, less wear and tear on other joints, and leisure activities (vacation, social activities, attending events) [8]. One could argue that these themes could not be addressed by TKA surgery alone. Nevertheless, when counselling patients, surgeons tend to focus on functional aspects like ROM, stability or alignment. Because of their proven relevance to patients, general health-related aspects should equally be discussed with patients.

The 31-item expectation questionnaire asked patients about exceeded expectations for the first time. 40% of patients responded that overall their expectations were better than expected. This is an important finding, as previous studies generally emphasised residual symptoms and impairments, and unfulfilled expectations more than positive outcomes. Exceeded expectations were previously determined by a pre- and post-op comparison of each item of the HHS-KRES [34]. The highest proportion of exceeded expectations in the presented study was seen for relief of knee pain (15%). Via pre- and post-op comparison, Tilbury et al. reported 22% of patients with exceeded pain relief [34]. It is important to acknowledge that patient expectations can be exceeded.

Group comparison revealed that not fully satisfied patients had significantly lower PROMs and lower ROM postoperatively. Interestingly, physical function, longer standing, climbing stairs and physical activities were least fulfilled in both satisfaction groups, but to considerably different proportions. Very satisfied patients showed excellent OKS scores postoperatively, but these particular expectations do not seem to be adequately reflected by this PROM. On the contrary, quality of life was fulfilled or exceeded in only 28% of not fully satisfied patients, whilst in 85% of very satisfied patients. This difference was reproduced in the postoperative EuroQol Index and VAS. Poor pre- and postoperative PROMs [13], unfulfilled expectations regarding physical activities [8, 35], as well as poor ability to perform knee-intensive activities requiring high flexion (e.g. stair climbing, gardening, dancing, squatting) [27, 28] were reported to be correlated with dissatisfaction. In terms of postoperative ROM, the logistic regression model showed a significant association with being very satisfied, but not the PROMs. In addition, very satisfied patients indicated that their expectations regarding ROM were fulfilled best. It could be argued that patients’ expectations concerning knee-intensive activities are too optimistic, and artificial joints are not designed to provide it. The results of the presented study indicate that patients have high expectations of ROM and its fulfilment contributes significantly to satisfaction.

The logistic regression model showed further the positive association in particular for exceeded and fulfilled main goals and for overall fulfilment. The relationship between fulfilled expectations and satisfaction in TKA has been extensively reported before [14]. Given the significant association between main goals and satisfaction, surgeons should ask patients about mandatory expectations when counselling on the surgery.

The considerable number of not fully satisfied patients, nearly 30% in this study, raises the question of how satisfaction should be measured. This has been very inconsistent and to date no gold standard exists, making comparisons difficult [19]. The cut-off point proposed by Tolk et al. should be critically examined and not fully satisfied patients should not be equated with dissatisfied patients. A validated cut-off point to distinguish between satisfied and not satisfied patients based on the NRS is needed. Despite advances in knee implants, surgical techniques, and pre-, peri-, and postoperative management in the last decades, numbers of dissatisfied patients remain at the same levels [8, 16, 37]. Unrealistic expectations can contribute to dissatisfaction and should therefore be addressed before surgery [2, 14]. Setting realistic expectations, either by the surgeon reflecting on the likelihood of achieving them or by modifying patient expectations via educational interventions, has been proposed by several studies [8, 12, 16, 22, 31, 34, 36]. The results of the presented study suggest that surgeons counselling for TKA should specifically include expectations that are individually mandatory for successful TKA as well as general health aspects and knee-intensive activities requiring high ROM.

This study has some limitations. The used expectation questionnaire was not tested for its measurement properties and unknown problems e.g. in terms of construct validity or responsiveness could have biassed the results. Furthermore, the comprehensive questionnaire was combined with several other PROMs, which might have influenced acceptance and filling in by patients. However, with only 33 not completed follow-ups, there was a good response rate. Discrimination of satisfaction groups has no methodical validation [36].

## Conclusion

A set of 17 key expectations for successful TKA was found, with equal emphasis on knee-related and general health-related aspects. Their fulfilment was positively associated with satisfaction. The highest fulfilment was seen in the relief of knee pain, lowest in physical function, and overall fulfilment of expectations was exceeded in 40% of patients. Not fully satisfied patients had lower PROMs and higher proportions of not and only partially fulfilled expectations. To avoid unrealistic expectations, surgeons need to ask patients’ mandatory expectations for successful TKA and counsel them about the likelihood of their fulfilment.

## Supplementary Information

Below is the link to the electronic supplementary material.

Supplementary file1 (DOCX 45 KB)

## Data Availability

Data are not publicly available.

## Abbreviations

ASA: American Society of Anesthesiologists
BMI: Body mass index
HHS-KRES: Hospital for special surgery knee replacement expectation survey
NRS: Numeric rating scale
OA: Osteoarthritis
OKS: Oxford knee score
PROMs: Patient-reported outcome measures
Q: Quartile (Q1–25% Quartile; Q3–5% Quartile)
ROM: Range of motion
SD: Standard deviation
THA: Total hip arthroplasty
TKA: Total knee arthroplasty
UCLA: University of California, Los Angeles
VAS: Visual analogue scale
vs.: Versus
