# Supplementary material for: Fulfilment of patients’ mandatory expectations are crucial for satisfaction: a study amongst 352 patients after total knee arthroplasty (TKA)
Source: Knee Surg Sports Traumatol Arthrosc. 2023 Feb 6;31(9):3755–64. doi: 10.1007/s00167-022-07301-y (PMC10435619; doi:10.1007/s00167-022-07301-y)
Supplement: Supplementary file 1 — Supplementary file1 (DOCX 45 KB) [file 167_2022_7301_MOESM1_ESM.docx]

**Expectation Questionnaire**

To what extent were your expectations met regarding the following topics?

Please determine whether the expectations stated below were **exceeded**, **fulfilled**, **partially fulfilled** or **not fulfilled**. If you didn’t have a certain expectation, please choose **not applicable**.

If in doubt, please mark the most applicable answer. Please note that you can only mark one answer per question

| **Symptoms** |  |  |  |  |  |
| --- | --- | --- | --- | --- | --- |
|  | **exceeded** | **fulfilled** | **partially fulfilled** | **not fulfilled** | **not applicable** |
| Relieved knee pain | □ | □ | □ | □ | □ |
| Reduced noises in knee joint | □ | □ | □ | □ | □ |
| Reduced swelling of knee joint | □ | □ | □ | □ | □ |
| **Physical functions** |  |  |  |  |  |
|  | **exceeded** | **fulfilled** | **partially fulfilled** | **not fulfilled** | **not applicable** |
| Improved range of motion of knee | □ | □ | □ | □ | □ |
| Improved muscle strength of leg | □ | □ | □ | □ | □ |
| Improved knee stability | □ | □ | □ | □ | □ |
| Straight leg (alignment) | □ | □ | □ | □ | □ |
| Improved physical endurance | □ | □ | □ | □ | □ |
| **Physical activity** |  |  |  |  |  |
|  | **exceeded** | **fulfilled** | **partially fulfilled** | **not fulfilled** | **not applicable** |
| Improved physical functions  (e.g. sit down, kneel down, long standing) | □ | □ | □ | □ | □ |
| Improved walking distance  (being able to walk a certain distance) | □ | □ | □ | □ | □ |
| Improved climbing stairs | □ | □ | □ | □ | □ |
| Improved physical activities  (e.g. gardening, cycling) | □ | □ | □ | □ | □ |
| Improved sport activities | □ | □ | □ | □ | □ |
| Improved sexual activities | □ | □ | □ | □ | □ |
| Improved longer standing | □ | □ | □ | □ | □ |

| **Quality of life** |  |  |  |  |  |
| --- | --- | --- | --- | --- | --- |
|  | **exceeded** | **fulfilled** | **partially fulfilled** | **not fulfilled** | **not applicable** |
| Improved quality of life | □ | □ | □ | □ | □ |
| Improved general health status  (fully physical, psychological and social well-being and not only the absence pf disease or disability) | □ | □ | □ | □ | □ |
| Improved participation in social life (e.g. get-together with family, friends) | □ | □ | □ | □ | □ |
| Undisturbed sleep | □ | □ | □ | □ | □ |
| **Coping strategies** |  |  |  |  |  |
|  | **exceeded** | **fulfilled** | **partially fulfilled** | **not fulfilled** | **not applicable** |
| Independence from others | □ | □ | □ | □ | □ |
| Independence from walking aids  (e.g. crutches or walkers) | □ | □ | □ | □ | □ |
| Reduced regular medication and possible side-effects | □ | □ | □ | □ | □ |
| **Activities of daily life** |  |  |  |  |  |
|  | **exceeded** | **fulfilled** | **partially fulfilled** | **not fulfilled** | **not applicable** |
| Improved performing of daily activities (e.g. grocery shopping, putting on stockings, use of public transport) | □ | □ | □ | □ | □ |
| Improved ability to work | □ | □ | □ | □ | □ |
| Improved performing of household tasks | □ | □ | □ | □ | □ |
| Improved mobility  (e.g. use of car, train, bus, bicycle) | □ | □ | □ | □ | □ |
| Improved performing of personal hygiene | □ | □ | □ | □ | □ |
| **Various issues** |  |  |  |  |  |
|  | **exceeded** | **fulfilled** | **partially fulfilled** | **not fulfilled** | **not applicable** |
| Prevention secondary impairments  (e.g. overloading the other leg, back pain) | □ | □ | □ | □ | □ |
| Weight reduction due to improved physical activity | □ | □ | □ | □ | □ |
| Longevity of implant  (time until revision surgery is necessary) | □ | □ | □ | □ | □ |
| Short hospital stay | □ | □ | □ | □ | □ |

| **Overall fulfillment of expectations** |
| --- |

Please indicate on the scale to which extend your expectations were fulfilled or even exceeded by marking it with a cross.


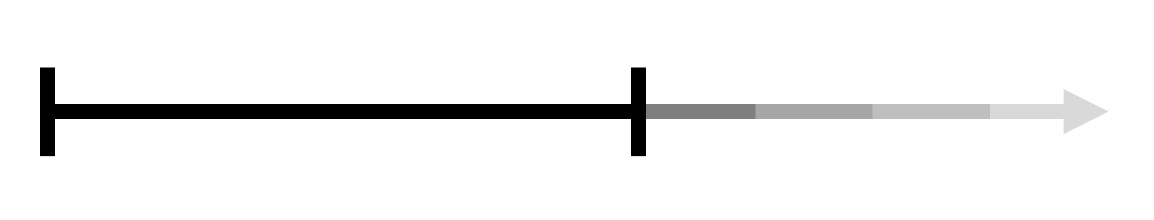


**Overall,**

**expectations exactly fulfilled**

**Overall,**

**expectations not fulfilled at all**

**Overall,**

**expectations exceeded**
